# The WHO interim Medical Countermeasures Network for public health emergencies

**DOI:** 10.2471/BLT.26.295961

**Published:** 2026-10-08

**Authors:** Tim Nguyen, Oluwatosin Wuraola Akande, Emanuele Bruni, David Manuel Cuenca, Reena H Doshi, Daniel Eibach, Ioana Ghiga, Shoshanna Goldin, Alexandra Hill, Qiu Yi Khut, John Lee, Ramona Ludolph, Constance McDonough-Thayer, Paul Molinaro, Murat Hakan Ozturk, Edwin Ceniza Salvador, Laetitia Sieffert, Lorenzo Subissi, Pushpa Ranjan Wijesinghe, Maria Van Kerkhove

**Affiliations:** a Department of Epidemic and Pandemic Management, Health Emergencies Preparedness and Response Programme, World Health Organization (WHO), Avenue Appia 20, 1211 Geneva, Switzerland.; b WHO Health Emergencies Programme Emergency Operations, WHO Regional Office for Europe, Copenhagen, Denmark.; c Health Emergency Preparedness and Response Programme, WHO Regional Office for the Eastern Mediterranean, Cairo, Egypt.; d Emergency Preparedness and Response, WHO Regional Office for Africa, Dakar, Senegal.; e Health Emergencies Preparedness and Response Programme, WHO Regional Office for the Western Pacific, Manila, Philippines.; f Sydney, Australia.; g Geneva, Switzerland.; h Health Emergency Alert and Response Operations Department, WHO, Geneva, Switzerland.; i Regional Revolving Funds, WHO Regional Office for the Americas, Washington DC, United States of America.; j Office of the WHO Representative, WHO Country Office, Kabul, Afghanistan.; k Science Division, WHO, Geneva, Switzerland.; l Health Emergencies Preparedness and Response Programme, WHO Regional Office for South-East Asia, New Delhi, India.

## Abstract

Medical countermeasures, such as vaccines, diagnostics and therapeutics, are essential for effectively preparing for and responding to public health emergencies. The coronavirus disease 2019 pandemic and other emergencies, including the mpox and Ebola outbreaks, exposed deep-seated inequities and fragmentation in the global system for accessing and distributing medical countermeasures. In 2023, the World Health Organization (WHO) established the interim Medical Countermeasures Network following a call from Member States for a coordination mechanism to facilitate access to medical countermeasures in the event of another pandemic. The network’s aim is to enhance timely and equitable access to quality, safe, effective and affordable medical countermeasures during public health emergencies. Operating as an interim network of networks, the interim Medical Countermeasures Network brings together global and regional stakeholders across the medical countermeasures value chain, without pre-empting Member States’ prerogatives during deliberations on the WHO Pandemic Agreement. The network’s value was demonstrated in 2024 during the mpox public health emergency of international concern, when its structure was leveraged to operationalize an Access and Allocation Mechanism for limited vaccine supplies. Through coordinated partnerships, the mechanism enabled the strategic sourcing, allocation, transportation and distribution of 1.49 million mpox vaccine doses to beneficiaries in 15 countries. The experience offered valuable insights for preventing, preparing for and responding to future pandemics. The experience also highlighted the need for a sustainable, institutionalized mechanism to coordinate equitable access to medical countermeasure that is aligned with global instruments including the *International health regulations* (2005) and the WHO Pandemic Agreement, once it comes into force.

## Résumé

Les contre-mesures médicales, telles que les vaccins, les tests de dépistage et les traitements, sont essentielles pour se préparer et réagir efficacement aux urgences de santé publique. La pandémie de maladie à coronavirus 2019 (COVID-19) et d’autres urgences, notamment les épidémies de mpox et d’Ebola, ont mis en évidence des inégalités profondément enracinées ainsi qu’une fragmentation du système mondial d’accès et de distribution des contre-mesures médicales. En 2023, l’Organisation mondiale de la Santé (OMS) a mis en place le Réseau intérimaire de contre-mesures médicales à la suite d’un appel lancé par les États membres en faveur d’un mécanisme de coordination visant à faciliter l’accès aux contre-mesures médicales en cas de nouvelle pandémie. L’objectif de ce réseau est d’améliorer l’accès rapide et équitable à des contre-mesures médicales de qualité, sûres, efficaces et abordables lors des urgences de santé publique. Fonctionnant comme un réseau provisoire de différents réseaux, le Réseau intérimaire de contre-mesures médicales rassemble des acteurs mondiaux et régionaux tout au long de la chaîne de valeur des contre-mesures médicales, sans empiéter sur les prérogatives des États membres lors des délibérations au sujet de l’Accord de l’OMS sur les pandémies. L’intérêt de ce réseau s’est confirmé en 2024 lors de l’urgence de santé publique d’importance internationale liée à la mpox, lorsque sa structure a été mise à profit pour mettre en œuvre un mécanisme d’accès et d’allocation destiné aux stocks limités de vaccins. Grâce à des partenariats coordonnés, ce mécanisme a permis l’approvisionnement stratégique, l’allocation, le transport et la distribution de 1,49 million de doses de vaccin contre la mpox dans 15 pays. Cette expérience a fourni des renseignements précieux en matière de prévention, de préparation et de réaction à de futures pandémies. Elle a également mis en évidence la nécessité d’un mécanisme durable et institutionnalisé permettant de coordonner l’accès équitable aux contre-mesures médicales, conformément aux instruments mondiaux tels que le *Règlement sanitaire international* (2005) et l’Accord de l’OMS sur les pandémies, dès son entrée en vigueur.

## Resumen

Las contramedidas médicas, como las vacunas, los medios de diagnóstico y los tratamientos, son esenciales para prepararse y responder eficazmente ante las emergencias de salud pública. La pandemia de enfermedad por coronavirus de 2019 (COVID-19) y otras emergencias, incluidos los brotes de mpox y de enfermedad por el virus del Ébola, pusieron de manifiesto profundas inequidades y la fragmentación del sistema mundial de acceso a las contramedidas médicas y su distribución. En 2023, la Organización Mundial de la Salud (OMS) estableció la Red provisional de contramedidas médicas, atendiendo al llamamiento de los Estados Miembros para crear un mecanismo de coordinación que facilitara el acceso a las contramedidas médicas en caso de una nueva pandemia. El objetivo de la red es mejorar el acceso oportuno y equitativo a contramedidas médicas de calidad, seguras, eficaces y asequibles durante las emergencias de salud pública. Concebida como una red provisional de redes, la Red provisional de contramedidas médicas reúne a partes interesadas mundiales y regionales de toda la cadena de valor de las contramedidas médicas, sin prejuzgar las prerrogativas de los Estados Miembros durante las deliberaciones acerca del Acuerdo de la OMS sobre Pandemias. La utilidad de la red quedó demostrada en 2024, durante la emergencia de salud pública de importancia internacional por mpox, cuando se aprovechó su estructura para poner en funcionamiento un Mecanismo de Acceso y Asignación ante la disponibilidad limitada de vacunas. Mediante alianzas coordinadas, el mecanismo permitió la adquisición estratégica, la asignación, el transporte y la distribución de 1,49 millones de dosis de vacunas contra la mpox a personas beneficiarias de 15 países. La experiencia aportó enseñanzas valiosas para la prevención, la preparación y la respuesta ante futuras pandemias. La experiencia también destacó la necesidad de contar con un mecanismo sostenible e institucionalizado que coordine el acceso equitativo a las contramedidas médicas y esté armonizado con instrumentos mundiales, entre ellos el *Reglamento Sanitario Internacional* (2005) y el Acuerdo de la OMS sobre Pandemias, una vez que este último entre en vigor.

## ملخص

تُعد التدابير الوقائية الطبية، مثل اللقاحات ووسائل التشخيص والعلاجات، عناصر أساسية للاستعداد الفعال لحالات الطوارئ الصحية العامة والاستجابة لها. وقد كشفت جائحة مرض كوفيد 19 وحالات طوارئ أخرى، بما في ذلك تفشي جدري القردة والإيبولا، عن أوجه تفاوت متجذرة وتشتت في النظام العالمي لإتاحة التدابير الوقائية الطبية وتوزيعها. وفي عام 2023، أنشأت منظمة الصحة العالمية "الشبكة المؤقتة للتدابير الوقائية الطبية" بعد دعوة الدول الأعضاء لإيجاد آلية تنسيقية لتيسير الحصول على التدابير الوقائية الطبية في حالة حدوث جائحة أخرى. وهدف الشبكة هو تعزيز فرص الحصول في الوقت المناسب وبشكل عادل على تدابير وقائية طبية تتسم بالجودة والأمان والفعالية والتكلفة المقبولة أثناء حالات الطوارئ الصحية العامة. وتعمل هذه الشبكة بوصفها شبكة للشبكات، حيث أن الشبكة المؤقتة للتدابير الوقائية الطبية تجمع معًا الأطراف المعنية العالمية والإقليمية العاملة في سلسلة القيمة الخاصة بالتدابير الوقائية الطبية، وذلك دون المساس بصلاحيات الدول الأعضاء أثناء مداولات اتفاقية منظمة الصحة العالمية بشأن الجوائح. وقد ظهرت قيمة الشبكة في عام 2024 أثناء حالة الطوارئ الصحية العامة التي تثير قلقاً دولياً والمتعلقة بجدري القردة، حيث تمت الاستفادة من هيكلها لتفعيل آلية للوصول والتخصيص للإمدادات المحدودة من اللقاحات. ومن خلال حالات شراكة منسقة، أتاحت هذه الآلية تدبير الإمدادات استراتيجياً وتخصيصها ونقلها وتوزيع 1.49 مليون جرعة من لقاح جدري القردة على المستفيدين في 15 دولة. وقد قدمت هذه التجربة تحليلات ذات قيمة فيما يتعلق بالوقاية من الجوائح المستقبلية والاستعداد لها والتعامل معها. كما سلطت التجربة الضوء على الحاجة إلى آلية مستدامة ومؤسسية لتنسيق الوصول العادل للتدابير الوقائية الطبية، بحيث تكون متماشية مع الأدوات العالمية بما في ذلك *اللوائح الصحية الدولية * (2005) واتفاقية منظمة الصحة العالمية بشأن الجوائح، بمجرد دخولها حيز التنفيذ.

## 摘要

疫苗、诊断工具和治疗药物等医疗对策对于有效防范和应对突发公共卫生事件至关重要。2019 冠状病毒病大流行以及包括猴痘和埃博拉疫情在内的其他突发事件暴露了全球医疗对策可及与分配系统中根深蒂固的不平等和碎片化问题。会员国呼吁制定协作机制，以便在再次出现大流行之时促进医疗对策获取；世界卫生组织（世卫组织）于 2023 年应其要求构建了临时医疗对策网络。该网络旨在加强突发公共卫生事件期间及时公平地获取优质、安全、有效且可负担的医疗对策的可及性。作为一个临时性的网络之网络，临时医疗对策网络汇聚了医疗对策价值链上的全球和区域利益相关方，且不妨碍会员国行使其在审议《世卫组织大流行协定》过程中的各项特权。当 2024 年猴痘疫情构成国际关注的突发公共卫生事件时，该网络的价值得到了证明：其架构被用于投入运行针对有限疫苗供应的获取和分配机制。通过协同的伙伴关系，该机制实现了战略筹措、分配、运输和分发 149 万剂猴痘疫苗，惠及 15 个国家的受益人。该经验为预防、防范和应对未来大流行提供了宝贵见解。该经验还凸显了有必要建立可持续的制度化机制，以协调医疗对策的公平获取，并与全球性文书（包括《国际卫生条例》(2005) 和生效后的《世卫组织大流行协定》）保持一致。

## Резюме

Медицинские контрмеры, такие как вакцинация, обеспечение средствами диагностики и лекарственными препаратами, имеют важнейшее значение для эффективной подготовки к чрезвычайным с точки зрения общественного здравоохранения ситуациям и реагирования на них. Пандемия коронавирусной инфекции 2019 года (COVID-19) и другие чрезвычайные ситуации, включая вспышки оспы обезьян и болезни, вызванной вирусом Эбола, выявили глубоко укоренившееся неравенство и разобщенность глобальной системы обеспечения доступа к медицинским контрмерам и их распределения. В 2023 году Всемирная организация здравоохранения (ВОЗ) учредила временную Сеть по медицинским контрмерам в ответ на призыв государств-членов создать координационный механизм для облегчения доступа к медицинским контрмерам на случай возникновения новой пандемии. Цель сети – расширить своевременный и справедливый доступ к качественным, безопасным, эффективным и доступным по цене медицинским контрмерам в период чрезвычайных с точки зрения общественного здравоохранения ситуаций. Действуя как переходная «сеть сетей», временная Сеть по медицинским контрмерам объединяет глобальных и региональных участников на всех этапах цепочки создания ценности медицинских контрмер, не предвосхищая и не ущемляя прерогатив государств-членов в ходе обсуждений Соглашения ВОЗ по борьбе с пандемиями. Ценность сети была продемонстрирована в 2024 году во время чрезвычайной с точки зрения общественного здравоохранения ситуации, имеющей международное значение, связанной со вспышкой оспы обезьян, когда ее структура была задействована для запуска механизма по обеспечению доступа и распределению в условиях ограниченных поставок вакцин. Благодаря скоординированному партнерскому взаимодействию данный механизм обеспечил стратегическое приобретение, распределение, транспортировку и доставку 1,49 миллиона доз вакцины против оспы обезьян получателям в 15 странах. Полученный опыт позволил сделать ценные выводы для предотвращения пандемий в будущем, обеспечения готовности к ним и реагирования на них. Этот опыт также подчеркнул необходимость создания устойчивого институционализированного механизма координации справедливого доступа к медицинским контрмерам, согласованного с глобальными инструментами, включая *Международные медико-санитарные правила* (2005 г.) и Соглашение ВОЗ по борьбе с пандемиями после вступления последнего в силу.

## Introduction

Medical countermeasures, including vaccines, therapeutics, diagnostics and other health products, are critical for preventing, detecting and responding to infectious diseases.^1^ The coronavirus disease 2019 (COVID-19) pandemic underscored the importance of access to life-saving medical countermeasures for managing public health emergencies globally. Although scientific and technological breakthroughs enabled medical countermeasures, particularly vaccines and antivirals, to be developed rapidly during the pandemic, timely and equitable access was challenging, particularly for low- and middle-income countries and for places that had constrained resources or were affected by conflict.^2^ Moreover, inadequate cohesiveness across different initiatives and mechanisms internationally meant that the delivery of medical countermeasures was reactive and ad hoc, which revealed limitations in their scalability and coordination.^3^

In response to these challenges and building on the lessons learnt in tackling them, Member States of the World Health Organization (WHO) called for an inclusive consultative process to develop an interim coordination mechanism for facilitating global access to medical countermeasures. The proposal was discussed in several high-level global forums, including a side event on timely and equitable access to medical countermeasures at the 78th United Nations (UN) General Assembly,^4^ and led to the 2021 World Health Assembly resolution WHA74.7,^5^ the 2023 UN General Assembly’s political declaration on pandemic prevention, preparedness and response, and the 2023 Group of 20 health ministers outcome document.^6^^,^^7^ As a result, the WHO interim Medical Countermeasures Network was established in 2023.^8^

The interim Medical Countermeasures Network’s aim is to enhance timely and equitable access to safe, effective and affordable medical countermeasures during public health emergencies. The network was designed to function in the interim period before the entry into force of the WHO Pandemic Agreement, which was adopted by the World Health Assembly on 20 May 2025.^9^^,^^10^ As tasked by the World Health Assembly, the Intergovernmental Working Group on the Pandemic Agreement is now drafting and negotiating details of the Pathogen Access and Benefit Sharing system, which will form an annex to the Agreement.^11^ When this annex is adopted by the World Health Assembly, the full Agreement will be open for countries to sign and ratify according to their own constitutional processes.^12^ The Pandemic Agreement will officially enter into force 30 days after 60 countries have ratified it.

The network is guided by core principles to uphold equity, transparency, solidarity, inclusiveness and accountability in all aspects of its work, which involves generating actionable insights, delivering a measurable impact, navigating complex systems, leading with integrity, and fostering collaboration between stakeholders. In practice, the network has adopted a network-of-networks approach that builds on existing initiatives and facilitates collaboration across the global medical countermeasure value chain. The value chain includes fast-tracked research and development, scalable manufacturing, end-to-end health emergency supply chains, integrated delivery and sustainable financing (Fig. 1).

**Fig. 1:**
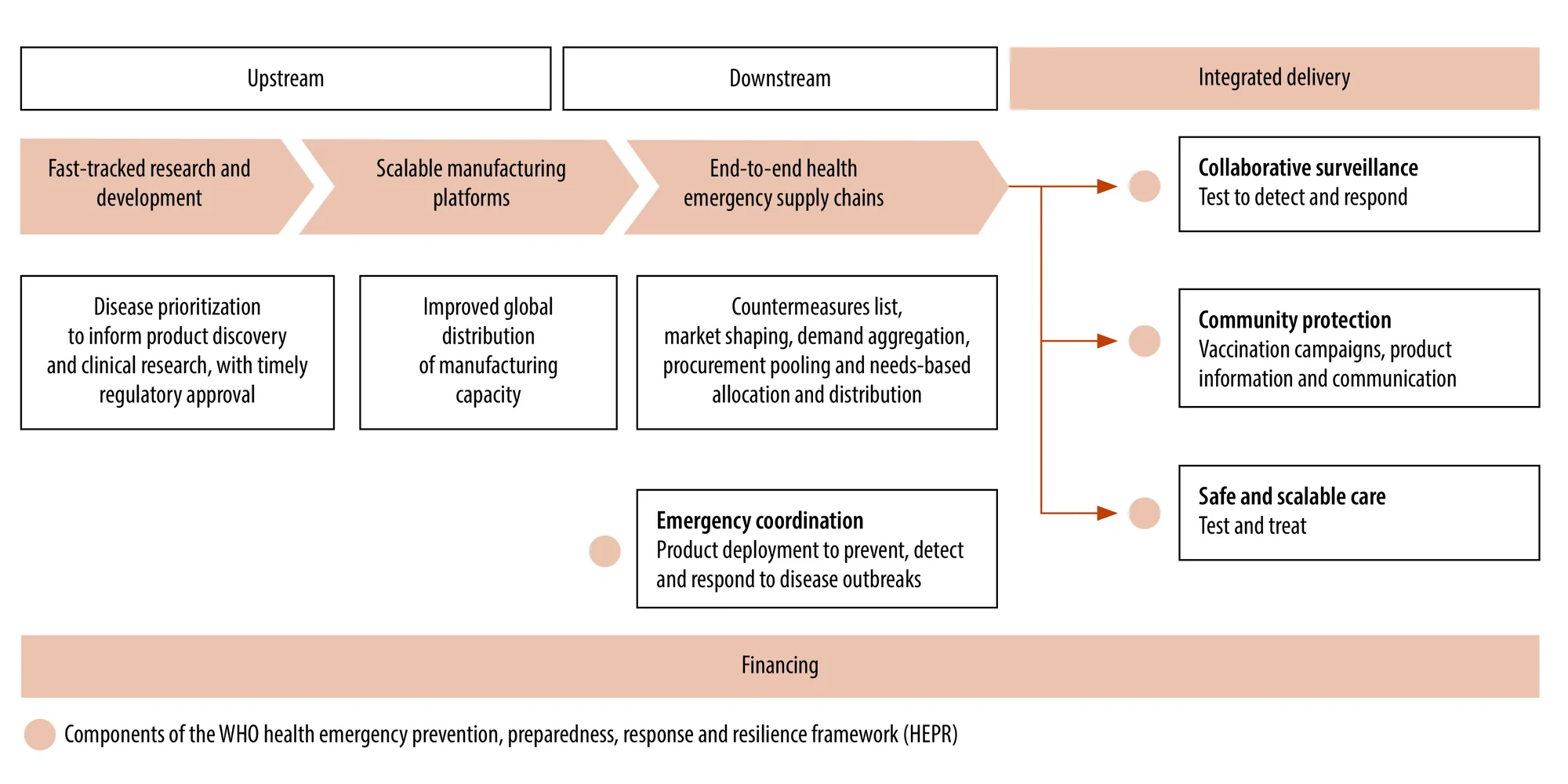
Access to medical countermeasures as one of the five components of WHO’s health emergency prevention, preparedness, response and resilience framework^13^

WHO: World Health Organization.

The network’s actions are aligned with commitments outlined in the amended version of the International Health Regulations (2005),^14^ which entered into force in September 2025,^15^ particularly commitments on removing barriers to equitable access to medical countermeasures during public health emergencies of international concern and pandemic emergencies. While Member States draft and negotiate the Pathogen Access and Benefit Sharing annex of the WHO Pandemic Agreement,^9^ the network is playing a strategic role in advancing global efforts to strengthen access to, and the coordination of, medical countermeasures during public health emergencies. The network is also helping to strengthen one of the core health emergency components (that is, access to countermeasures) of WHO’s global architecture for health emergency prevention, preparedness, response and resilience and to ensure that the architecture is systematically aligned with regional health emergency initiatives.^13^ Furthermore, the network’s actions are aligned with WHO’s 14th General Programme of Work,^16^ which emphasizes the need for more timely and equitable access to medical countermeasures. The network performs these roles by: (i) fostering collaboration across existing networks; (ii) mapping ongoing activities; (iii) promoting global coordination to create synergies and the efficient use of resources; and (iv) addressing gaps in the medical countermeasures’ value chain through collaborative action.

The aim of this paper is to document the establishment and early operationalization of the interim Medical Countermeasures Network as a critical global response mechanism for addressing systemic gaps in timely and equitable access to medical countermeasures in the period before WHO’s Pandemic Agreement enters into force. In addition, the paper discusses the network’s governance mechanisms, the lessons learnt from its operationalization, and the next steps to be taken in strengthening the medical countermeasures ecosystem as an integral component of health emergency prevention, preparedness, response and resilience.

## Establishing the network

The COVID-19 pandemic exposed profound vulnerabilities in the global health architecture that, in particular, affected the availability of, and equitable access to, medical countermeasures and other health products, such as personal protective equipment and biomedical products. During the pandemic, global access to medical countermeasures was constrained by limited capacity in research and development, manufacturing and procurement.^17^ Despite efforts to promote solidarity globally, the COVID-19 pandemic was marked by the inequitable distribution of medical countermeasures among countries, even after global supply constraints had eased in the later phase of the pandemic.^18^ There was a mismatch between global demand and production capacity, which was concentrated in a few manufacturing countries.^19^^,^^20^ Many countries, particularly low- and middle-income countries, faced persistent barriers to timely access to medical countermeasures, which highlighted structural inequities and fragmented coordination mechanisms in the global health system.^21^

Two major mechanisms established during the pandemic, the UN’s COVID-19 Supply Chain System and the Access to COVID-19 Tools Accelerator, achieved notable results but also revealed structural gaps in governance, financing and country engagement,^22^^–^^24^ as detailed in independent evaluations (Box 1).

Box 1 **Lessons from COVID-19 coordination mechanisms, 2020–2022**

In March 2020, during the early phase of the COVID-19 pandemic, the United Nations Crisis Management Team coordinated the establishment of the United Nations COVID-19 Supply Chain System. The supply chain system sought to consolidate demand, coordinate purchasing and ensure the delivery of essential supplies, such as personal protective equipment, diagnostics, therapeutics and biomedical equipment.^22^ In collaboration with global partners, the system enabled the procurement and distribution of commodities valued at about 1091 million United States dollars to 184 countries, with low- and middle-income countries receiving the majority of supplies by the end of 2020. Later, an independent assessment highlighted both the value of this rapid, collaborative mechanism and persistent challenges, for example, with financing, data visibility and market-tailored strategies for accessing critical supplies.^22^

In response to challenges with accessing medical countermeasures during the COVID-19 pandemic and drawing on lessons learnt from the 2009 A(H1N1) swine flu pandemic, the Access to COVID-19 Tools Accelerator was created in April 2020, less than three months after COVID-19 was declared a public health emergency of international concern.^23^ The aim of the Accelerator was to promote the development and production of, and equitable access to, essential COVID-19 health products.^24^ The Accelerator consisted of three vertical pillars: (i) diagnostics; (ii) therapeutics; and (iii) vaccines, access to which were coordinated by the COVID-19 Vaccines Global Access (COVAX) initiative.^25^ These were supported by a fourth pillar: the cross-cutting Health Systems and Response Connector pillar, whose aims were to strengthen national capacities and to enable health systems to implement new tools promptly and effectively when available.^23^ Building on these efforts, the COVID-19 Vaccine Delivery Partnership was launched in January 2022 to support vaccine delivery,^26^ with a focus on 34 countries. Notably, one of the initiatives that emerged from the Access to COVID-19 Tools Accelerator’s oxygen emergency task force was the Global Oxygen Alliance,^27^^,^^28^ which is a multistakeholder collaborative initiative for increasing access to oxygen in low- and middle-income countries.

An independent evaluation of the Access to COVID-19 Tools Accelerator, commissioned by the Access to COVID-19 Tools Accelerator Facilitation Council in July 2022, noted that the Accelerator was perceived as enabling unprecedented coordination among global health agencies at the time.^23^ The Accelerator provided dedicated support for countries trying to achieve the vaccination coverage levels recommended by WHO.^29^ Of the Accelerator’s four pillars, the vaccine pillar was seen as the most successful. Approximately 2 billion vaccine doses along with safe injection devices were delivered to 146 countries and 10 different COVID-19 vaccine products were shipped, thereby averting an estimated 2.7 million deaths.^23^^,^^25^ The Health Systems and Response Connector pillar, which was designed to strengthen links between the three vertical pillars and to support country health systems, was viewed as less successful. The Health Systems and Response Connector pillar faced implementation challenges and was described as largely ineffective in some areas; among other shortcomings, coordination was weak and funding was limited.^23^ The independent evaluation also noted limitations in the Accelerator’s operating model. For example, the ad hoc nature of the coordination mechanism presented challenges for sustainability and scalability, which could affect future pandemic responses, as did insufficient engagement by the governments of low- and middle-income countries. Other challenges identified included limited integration across the Accelerator’s four pillars, weak accountability structures, a lack of coordination among global and regional partners, and limited funding and resource mobilization. The evaluation provided important recommendations for future mechanisms,^23^ including (i) the establishment of a formal governance structure with better coordination; (ii) the creation of a pool of contingent funding to enable responses and commitments at day zero; (iii) the development and expansion of sustainable regional manufacturing capacity; and (iv) the continuous strengthening of health systems.

COVID-19: coronavirus disease 2019; WHO: World Health Organization.

Recognizing the lessons learnt and in response to calls from high-level fora, WHO embarked on a consultative process that led to the establishment of the interim Medical Countermeasures Network in 2023, with the goal of enhancing collaboration to ensure timely and equitable access to medical countermeasures during epidemics and pandemics.^8^ Building on experience, the network considered the recommendations of independent evaluations of mechanisms established during the COVID-19 pandemic: it set up a standing coordination architecture and secretariat ahead of the next emergency and adopted an end-to-end value-chain approach that connects research and development, manufacturing, procurement, delivery and financing through the Partners Forum, which is a WHO-hosted multistakeholder platform. The engagement of Member States was embedded in the governance of the network from the outset, and the network has responded to calls for day-zero funding to ensure that funding for securing access to medical countermeasures is available at the start of a health emergency.

## Governance and partnerships

The governance structure of the interim Medical Countermeasures Network was designed to balance strategic oversight with inclusive stakeholder participation, thereby ensuring that partner mandates are consistent and that organizational responses are sufficiently agile to adapt to each emergency and its specific needs, while at the same time aligning global objectives with regional and national priorities.

### Member State engagement

Member States’ sense of ownership of, and active engagement with, WHO’s interim Medical Countermeasures Network have been central to shaping the network as a mechanism that meets Member States’ priorities and builds on and leverages existing initiatives and networks. Between July 2023 and August 2024, seven structured consultations with Member States were convened and provided a consistent platform for dialogue, feedback and co-creation. Engagement was also facilitated through Member State-led processes, such as a side event on medical countermeasures at the 78th UN General Assembly,^4^ the Johannesburg process (co-chaired by Norway and South Africa) and G7 and G20 meetings,^30^^–^^32^ all of which helped shape global approaches to the development, production and equitable distribution of medical countermeasures.

Guided by these consultations, the network adopted a network-of-networks approach, an inclusive and collaborative operating model that leverages existing technical expertise, operational capacity and political will. This foundation ensures that the network remains responsive to public health emergencies while promoting coherence and coordination across the medical countermeasures value chain.

### Partner engagement

In line with its constitutional mandate as the directing and coordinating authority on health internationally,^33^ WHO plays a central role in convening and coordinating global efforts to ensure timely and equitable access to medical countermeasures.^4^^–^^7^ In particular, WHO fostered broad-based collaboration across the global health landscape to advance the vision of the interim Medical Countermeasures Network.

A broad consultative process initiated by WHO that involved UN agencies, global health and financing organizations, civil society and nongovernmental organizations, regional entities, industry and the private sector led to the establishment of the interim Medical Countermeasures Network Partners Forum, a WHO-hosted multistakeholder platform that included more than 50 organizations in October 2025 (Box 2). Since June 2023, this forum has facilitated structured engagement with partners, regional bodies, civil society organizations and industry representatives. To translate this engagement into coordinated action across the value chain, the forum established health product-specific workstreams: the vaccines workstream was launched in October 2025 and the diagnostics workstream was launched in May 2026. In addition, the interim Medical Countermeasures Network has begun exploring pooled regional procurement through two workshops with regional entities and a workshop on the sidelines of the Global Health Security Conference in June 2026.^34^

Box 2 **WHO interim Medical Countermeasures Network partner organizations,^8^ 2025**

**International and United Nations agencies**

Pan American Health Organization Revolving Fund for Access to Vaccines; The World Bank; United Nations Children's Fund; United Nations High Commissioner for Refugees; Unitaid; World Food Programme; World Health Organization.

**International health and development organizations**

Bill and Melinda Gates Foundation; Clinton Health Access Initiative; Coalition for Epidemic Preparedness Innovations; Drugs for Neglected Diseases initiative; Foundation for Innovative New Diagnostics; Gavi, the Vaccine Alliance; International Pandemic Preparedness Secretariat; Medicines Patent Pool; Pasteur Network; Regionalized Vaccine Manufacturing Collaborative; The Global Fund; Wellcome Trust; International Medical Corps.

**Civil society and nongovernmental organizations**

AIDS Vaccine Advocacy Coalition; East African Health Portal; International Committee of the Red Cross; International Federation of Red Cross and Red Crescent Societies; Médecins Sans Frontières; Pandemic Action Network; Platform for Access to COVID-19 Tools Accelerator Civil Society and Community Representatives; Third World Network.

**Regional bodies**

Africa Centres for Disease Control and Prevention; Association of Southeast Asian Nations; Caribbean Public Health Agency; European Commission; European Research Infrastructure on Highly Pathogenic Agents; Gulf Centre for Disease Prevention and Control.

**Industry and private sector**

Advanced Medical Technology Association; Biotechnology Innovation Organization; Developing Countries Vaccine Manufacturers Network International; Generic and Biosimilar Medicines Association; Global Diagnostic Imaging Healthcare IT and Radiation Therapy Trade Association; Global Medical Technology Alliance; International Federation of Pharmaceutical Manufacturers and Associations; International Generic and Biosimilar Medicines Association; Pharmaceutical Research and Manufacturers of America.

Note: this list does not include all partner organizations involved in the World Health Organization’s interim Medical Countermeasures Network.

The forum continues to serve as a key platform for engagement between partners and has fostered the close operational relationships essential for effective coordination during public health emergencies. Sustained engagement has been instrumental in developing key deliverables such as the global medical countermeasures landscape analysis and the forthcoming high-level implementation plan,^1^ while reinforcing a culture of transparency, shared accountability and inclusivity.

## Key deliverables

### Global landscape analysis

In collaboration with partners, between January and August 2024, WHO conducted a comprehensive medical countermeasures landscape analysis of readiness for priority pandemic threats, including pandemic influenza, novel coronaviruses and unknown high-threat pathogens (Pathogen X) across the medical countermeasures value chain.^1^ This global assessment mapped key actors, activities and initiatives and identified opportunities for enhanced coordination and collaboration for the development of, and access to, medical countermeasures. The analysis highlighted the need for sustained end-to-end investment in medical countermeasures across the value chain. Several priority actions were identified for increasing equitable access, including: (i) adopting a viral-family approach and an inclusive research and development framework for accelerating the development of broadly applicable medical countermeasures; (ii) expanding regional production capacity; (iii) coordinating demand aggregation, supply and pooled procurement; (iv) implementing equitable approaches to the allocation of medical countermeasures; (v) promoting integrated delivery, acceptance and uptake of medical countermeasures; and (vi) strengthening coordinated financing.

### High-level implementation plan

Building on the findings of the global medical countermeasures landscape analysis, the interim Medical Countermeasures Network began developing a high-level implementation plan in September 2024. The plan, which is being developed with partners, includes an operational roadmap for translating the network’s vision into concrete priority actions across the medical countermeasures value chain and health product-specific workstreams.

After its anticipated completion in late 2026, the high-level implementation plan will serve as a guiding framework for the activities of the interim Medical Countermeasures Network over the following two years, provided the WHO Pandemic Agreement has not entered into force. The plan will promote synergies among stakeholders, capitalize on their respective capacities, and facilitate coordinated action to accelerate the availability and equitable delivery of medical countermeasures where they are most needed. The plan will also support the operationalization of the network at a regional level through engagement with regional bodies in a way that capitalizes on existing partnerships, experience and strategies in individual regions, thereby addressing the needs and priorities of both regions and countries in a more coordinated and context-specific manner. At the same time, interactions with the interim Medical Countermeasures Network’s global network will foster the exchange of information on lessons learnt and provide opportunities for cross-regional collaboration.

## The mpox Access and Allocation Mechanism

The effectiveness of the interim Medical Countermeasures Network in supporting responses to public health emergencies was exemplified by the operationalization of the multipartner access and allocation mechanism for vaccines during the mpox public health emergency of international concern in 2024.^35^ Activated in response to the urgent need for medical countermeasures, the mechanism leveraged the interim Medical Countermeasures Network to coordinate a transparent and collaborative process for the timely sourcing, allocation and delivery of limited vaccine supplies to people in the countries most affected by the emergency.

At the onset of the mpox emergency, the global distribution of vaccines was iniquitous, primarily due to their high cost.^36^ The mechanism secured donations for vaccines, procured vaccines and established an equitable allocation mechanism to support targeted vaccination activities in affected countries. These accomplishments were achieved in cooperation with a coalition of interim Medical Countermeasures Network partners,^8^ including: (i) the Africa Centres for Disease Control and Prevention (Africa CDC); (ii) the Coalition for Epidemic Preparedness Innovations; (iii) the Foundation for Innovative New Diagnostics; (iv) Gavi, the Vaccine Alliance; (v) the European Union’s Health Emergency Preparedness and Response Authority; (vi) the United Nations Children’s Fund; and (vii) WHO.

The equitable vaccine distribution was defined as if populations and countries facing the highest risk of disease spread, as determined by a risk matrix in the mpox continental strategic preparedness and response plan,^37^ had priority access to scarce vaccines. Allocation decisions were transparent, evidence-informed and guided by each country’s readiness to implement a timely and safe vaccination campaign. An independent technical expert review committee was established to assess each country’s use of vaccines. The committee operationalized equitable vaccine distribution using an allocation framework that incorporated epidemiological risk, mpox transmission dynamics, the outbreak burden, each country’s readiness to deploy vaccines, and vaccine demand in the country. The technical expert review committee was guided by regional leaders belonging to the Africa CDC and the WHO Regional Office for Africa, who together established the continental Incident Management Support Team.^38^ This governance structure played a central role in shaping priorities, informing allocation decisions and ensuring that the access and allocation mechanism responded to locally identified needs and operational realities. Progress towards equity was measured using indicators such as: (i) the proportion of doses delivered to the low- and middle-income countries affected; (ii) the timeliness of vaccine allocations; and (iii) the extent to which the available supply reached priority populations in countries experiencing active outbreaks, as defined in the mpox continental strategic preparedness and response plan. The mpox Access and Allocation Mechanism increased equitable access to vaccines by pooling and by allocating scarce global supplies according to public health needs. Approximately 2.5 million doses were secured, 2.3 million doses were allocated to 15 countries through seven allocation rounds and more than 1.49 million doses were delivered, including 719 200 to the Democratic Republic of the Congo, the epicentre of the outbreak.

The access and allocation mechanism supported countries in estimating vaccine needs, identifying available supply and allocating doses in a manner designed to optimize public health outcomes. In addition to vaccine delivery, access and allocation mechanism partners also supported recipient countries in developing and implementing their national vaccination strategies, thereby ensuring that the doses available were effectively deployed through targeted activities.

Beyond vaccines, a coordinated effort was also undertaken to address the acute shortage of diagnostic tests at the beginning of the mpox public health emergency of international concern. A Diagnostics Consortium for mpox, which comprised six implementing partners co-led by the Africa CDC and WHO, was established to enhance equitable access to scarce diagnostic tests and to coordinate and provide an insight into country-level diagnostic activities and needs. Over its operational period, the Consortium delivered more than 337 000 diagnostic tests, associated reagents and consumables to more than 65 countries.

The mpox Access and Allocation Mechanism also laid the foundations for an emergency global stockpile of mpox vaccines,^39^ which involved addressing gaps in data, obtaining financial estimates, developing a sustainable financing model and designing a stockpile mechanism that is now managed by the International Coordination Group on Vaccine Provision.^40^ Moreover, the lessons learnt during the mechanism’s operation offered valuable insights into how timely access to, and the equitable distribution of, medical countermeasures, such as diagnostics and therapeutics, can be addressed in future emergencies.

## The Bundibugyo Ebola response 

In situations where no countermeasures yet exist, the role of the interim Medical Countermeasures Network begins upstream, in aligning research and development with access goals so that products are not developed in the absence of a path to equitable deployment. Stages that are often pursued separately must be linked, from research through to manufacturing, procurement and delivery. This approach is being tested in the current response to Ebola.^41^

The 2026 outbreak of Ebola disease caused by the Bundibugyo virus has highlighted the challenges of responding to a public health emergency in the absence of licensed vaccines, therapeutics and disease-specific diagnostics. In this case, the interim Medical Countermeasures Network brought major global health organizations together through regular partners briefings, principals meetings and workstream meetings, with the goal of aligning priorities and coordinating action across the medical countermeasures ecosystem. The focus was on accelerating the development, evaluation and future deployment of candidate vaccines, therapeutics and diagnostics. Discussions centred not only on advancing clinical trials for promising vaccine and therapeutic candidates, but also on preparing for access to these products once evidence of their effectiveness emerges, while recognizing that regulatory approval and large-scale distribution may require considerable time and resources.

To bridge the gap between product development and availability, partners have produced post-clinical-trial access roadmaps for candidate therapeutics and prophylactic products, with the aim of ensuring that countries affected by an outbreak can benefit from successful products as rapidly as possible. This work has involved addressing structural barriers to financing, procurement and deployment that have historically delayed access to medical countermeasures during outbreaks. In parallel, the network has been supporting the establishment of a diagnostics evaluation and validation platform for assessing emerging Bundibugyo virus-specific diagnostic tests under field conditions, thereby enabling a product’s effectiveness to be validated and informed procurement decisions to be made rapidly as new products enter the market. Together, these actions demonstrate how the interim Medical Countermeasures Network can connect research, access planning and operational deployment to prepare for equitable access even before a medical countermeasure has been fully licensed.

## Structural barriers and limitations

The interim Medical Countermeasures Network was designed to address coordination challenges exposed during the COVID-19 epidemic, but several structural barriers to equitable access remain and are beyond its control. Manufacturing capacity for vaccines, therapeutics and diagnostics is concentrated in a few countries and little standing or contingent financing is available at the onset of emergencies.^19^^,^^20^^,^^23^ By aggregating demand, pooling procurement and coordinating financing, the network can improve the efficiency and equity of access to the products available and can align partners around expanding regional production. However, it cannot itself build manufacturing capacity or hold day-zero funds, these depend on sustained investment by Member States, financing institutions and industry.

The network also operates under constraints of its own. As a voluntary network of networks without an independent legal mandate or binding authority over its partners, its capacity to compel timely action, enforce equitable allocation or guarantee supply rests on the alignment, goodwill and resources of participating organizations and Member States. The network’s own operations are sustained by time-limited, earmarked financial contributions from a small number of donor governments, which leave it exposed to shifts in donor priorities and to the wider contraction of global health financing.

## Sustaining momentum for equitable access

As outbreaks of infectious diseases with epidemic and pandemic potential become increasingly frequent and complex, the interim Medical Countermeasures Network and its mechanisms are supporting end-to-end preparedness for, and responses to, outbreaks across the medical countermeasures value chain in the interim period before the WHO Pandemic Agreement enters into force. These activities include managing the availability of supplies, forecasting demand, allocating products, coordinating shipments and distribution, and using effective and efficient deployment strategies at the country level.

Recently, amendments to the International Health Regulations (2005) were adopted by WHO Member States during the 77th World Health Assembly and began to enter into force for most countries in September 2025, and the historic WHO Pandemic Agreement was adopted during the 78th World Health Assembly in May 2025.^10^^,^^42^ Their adoption underscores the strategic alignment between international legal instruments and ongoing efforts to prepare for global pandemics. These instruments aim to remove barriers to timely and equitable access to essential health products following the declaration of, and during, public health emergencies of international concern (including pandemics) and to ensure access is based on public health risks and needs.

Conceived as a time-bound bridge rather than a permanent institution, the interim Medical Countermeasures Network provided an opportunity to test, in real emergencies, the coordination functions any lasting mechanism will require. In accordance with the WHO Pandemic Agreement, the Conference of the Parties will govern the Agreement’s implementation and could give direction to the secretariat established for the network as an interim mechanism for the period before the WHO Pandemic Agreement is ratified. The secretariat will remain flexible enough to accept such directions before a definitive decision is made on the longer-term fate of the network. Being anchored within WHO’s health emergency prevention, preparedness, response and resilience framework, the progress achieved through the network provides an important foundation for establishing a sustainable and scalable approach to greater coordination across the medical countermeasures value chain. In addition, the network fostered co-creation between WHO Member States and partners and provided a structured partnership infrastructure that is capable of supporting coherent action during health emergencies. Sustaining political commitment to, and shared investment in, equitable access to medical countermeasures is essential for building a safer, better prepared and more resilient global health system for all.
